# Manipulation of the Fascial System Applied During Acute Inflammation of the Connective Tissue of the Thoracolumbar Region Affects Transforming Growth Factor-β1 and Interleukin-4 Levels: Experimental Study in Mice

**DOI:** 10.3389/fphys.2020.587373

**Published:** 2020-12-03

**Authors:** Maria Elisa Duarte França, Larissa Sinhorim, Daniel Fernandes Martins, Robert Schleip, Nicolas A. M. M. Machado-Pereira, Gabriel Melo de Souza, Verônica Vargas Horewicz, Gilmar Moraes Santos

**Affiliations:** ^1^Posture and Balance Laboratory (LAPEQ), College of Health Sciences and Sports, Santa Catarina State University (UDESC), Florianópolis, Brazil; ^2^Neurosciences Experimental Laboratory (LANEX), Postgraduate Program in Health Sciences (PPGCS), University of Southern Santa Catarina, Palhoça, Brazil; ^3^Department of Sport and Health Sciences, Associate Professorship of Conservative and Rehabilitative Orthopedics, Technical University of Munich, Munich, Germany; ^4^Department for Medical Professions, DIPLOMA Hochschule Bad Sooden-Allendorf, Bad Sooden-Allendorf, Germany

**Keywords:** fascial system, inflammation, cytokines, mice, manipulation

## Abstract

Fascia can become rigid and assume a fibrotic pattern due to inflammatory processes. Manipulation of the fascial system (MFS), manual technique targeting connective tissues, is commonly used in clinical practice in pain management. We aimed to verify MFS effects on the connective tissue inflammatory changes in mice. Swiss *Mus musculus* male mice (*n* = 44) were distributed into groups: carrageenan without treatment (Car, *n* = 11), carrageenan with MFS (Car + MFS, *n* = 12), saline without treatment (*n* = 10), and saline with MFS (saline + MFS, *n* = 11). Interleukin 4 (IL-4), IL-6, tumor necrosis factor (TNF), transforming growth factor β1 (TGF-β1), and monocyte chemoattractant protein 1 (MCP-1) levels were verified by enzyme-linked immunosorbent assay. Neutrophil (Ly-6G), macrophage (F4/80), and nitric oxide synthase 2 (NOS-2) were identified using Western blot. The MFS protocol was applied from the first to the third day after inflammation of the connective tissue of the thoracolumbar region. There was a significant MFS effect on IL-4 (*p* = 0.02) and TGF-β1 (*p* = 0.04), without increasing MCP-1, TNF, and IL-6 levels (*p* > 0.05) on thoracolumbar region from Car + MFS, in comparison with saline. Ly-6G in Car + MFS presented lower levels when compared with saline (*p* = 0.003) or saline + MFS (0.003). NOS-2 levels were lower in Car + MFS than in saline + MFS (*p* = 0.0195) or saline (*p* = 0.003). MFS may have an anti-inflammatory effect, based on TGF-β1 and IL-4. IL-4 may have inhibited neutrophil migration. Lower levels of NOS-2 may be linked to the lack of macrophages, which are responsible for NOS-2 expression.

## Introduction

Fascia is a connective tissue composed of collagen fibers (Willard et al., 2012) that enables sliding between surfaces (Langevin et al., 2011); it is known that it has an important role in locomotion withal, as it acts as a transmission force mechanism between adjacent synergist and antagonistic muscles (Garfin et al., 1981). It has been suggested that this transmission force mechanism occurs via collagen fibers of the connective tissue (Huijing and Baan, 2008), which shares the mechanical stress during muscle contraction (Findley et al., 2015). Moreover, the fascia resistance to pressure and traction depends on its extracellular matrix (ECM) content (Bhattacharya et al., 2010), which can be affected by inflammatory processes (Langevin et al., 2011).

Persistent inflammation processes can cause fibrosis and change mechanical properties of the connective tissue, which occurs when the main type of cells of the connective tissue, the fibroblasts (Langevin et al., 2011), proliferates (Morikawa et al., 2016) and differentiates into myofibroblasts (Morikawa et al., 2016; Pardali et al., 2017). Thereby, the stiffness of the connective tissue may affect sliding between surfaces, once the adjacent connective tissue layers are particularly relevant in structures, such as the connective tissue of the thoracolumbar region, in which the density layers correspond to aponeuroses of muscles with different traction directions: longitudinal (for latissimus dorsi, posterior serratus, and erector spinae) vs. transversal (for internal/external obliques and latissimus dorsi) (Langevin et al., 2011). If sliding between these tissues is compromised, and the fascia biomechanical properties are changed, it may contribute to musculoskeletal pain (Klingler et al., 2014).

In the early stages of acute inflammation, inflammatory macrophages type 1 (M1) are polarized by proinflammatory mediators, such as tumor necrosis factor (TNF) and interleukin 6 (IL-6), whereas in later stages, anti-inflammatory macrophages type 2 (M2) are stimulated by transforming growth factor β (TGF-β) and IL-4 (Fraternale et al., 2015). In addition, in the presence of IL-4, phagocytic cells, such as M1 macrophages, differentiate into the M2 phenotype (Mantovani et al., 2004). However, in chronic inflamed connective tissue, stiffness and fibrosis have been associated with an excess of TGF-β1 (Bouffard et al., 2008; Bove et al., 2016; Gyorfi et al., 2018) and IL-4 (Trautmann et al., 1998, 2000), as these mediators stimulate fibroblast activity (Trautmann et al., 2000) and their differentiation into myofibroblasts (Arora et al., 1999).

As a treatment for orthopedic conditions, such as stiffness and fibrosis, the manipulation of the fascial system (MFS) is commonly used (McKenney et al., 2013; Ajimsha et al., 2014). It is a manual application of a directional load (Ajimsha et al., 2014), aiming to gradually mobilize the soft tissue (McKenney et al., 2013; Rubira and Sousa, 2013) promoting mechanotransduction, mechanism by which cells convert mechanical stimuli into a chemical response (Schleip et al., 2005). The therapist is guided by the response of the patient body to determine the direction of the load and its intensity and duration (McKenney et al., 2013). The objective of the MFS is to restore optimal tension, reduce pain, and improve the myofascial complex function (Ajimsha et al., 2014).

However, MFS is poorly understood, and its effects on early inflammatory mediators of fibrotic processes have not been evaluated. In some experimental studies, such as those of Bove et al. (2016, 2019), and Altomare and Monte-Alto-Costa (2018), the effects of manual therapy in reducing tissue changes provoked by inflammation have been investigated. After the application of manual protocols, those authors have reported lower collagen type I and TGF-β1 levels in connective tissue (Bove et al., 2016), decreased neutrophils and macrophage cells and collagen deposition around the median nerve (Bove et al., 2019), lower presence of macrophages, and reduction of subcutaneous tissue stiffness (Altomare and Monte-Alto-Costa, 2018). Those studies evaluated a combination of joint mobilization and traction of skin tissue of the upper extremity in rats (Bove et al., 2016, 2019), or scar tissue mobilization (Altomare and Monte-Alto-Costa, 2018). None of those studies looked at the isolated effects of MFS on inflammatory mediators. Moreover, those studies investigated manual therapy after chronic changes in tissues. Important inflammatory mediators, such as TNF and IL-6, are present in the early phases of inflammation (Loram et al., 2007b; Annamalai and Thangam, 2017).

In the present study, using a translational research perspective, we investigated the effects of MFS on the connective tissue of the thoracolumbar in acute inflammation using a carrageenan-induced inflammation mice model. The objective of this study was to investigate the effects of MFS on inflammatory mediators and cells after induction of inflammation in the connective tissue of the thoracolumbar region with carrageenan in mice. We hypothesized that MFS would be a safe manual technique to be applied in early stages of inflammation to help inflammatory pain management in orthopedic conditions.

## Materials and Methods

This preclinical study was conducted by a partnership between Posture and Balance Laboratory at Santa Catarina State University and the Neurosciences Experimental Laboratory (LANEX) at the University of Southern of Santa Catarina (UNISUL), Brazil. The research was conducted at LANEX.

### Ethics Approval

This research was approved by the Ethics Commission for the Use of Animals (CEUA-Unisul), accredited by the National Council of Animal Experimentation. The research was registered using the protocol 18.017.4.08. IV (CEUA-UNISUL). The animals were treated in accordance with the ethical principles established by the Brazilian College of Animal Experimentation and the experiments followed the ARRIVE (animal research: reporting *in vivo* experiments) (Kilkenny et al., 2010).

### Animals

Animals were bred and provided by the Federal University of Santa Catarina and reallocated at LANEX/UNISUL. This study was carried out using Swiss *Mus musculus* male mice (*n* = 44), weighing 45–58 g, aged 8–14 weeks. The mice were randomly distributed into four groups, as described below. Each group was housed in a polypropylene box at LANEX/UNISUL, at an average temperature of 21–23°C, under a 12/12-h light–dark cycle, having free access to water and food. The sample size was based on *n* = {[(z alfa + z beta) × s]/sigma}^2^ formula (Wayne and Cross, 2018) and on experimental data of our laboratory.

### Groups

Animals were randomized into four groups: Carrageenan without treatment (Car, *n* = 11), Carrageenan treated using MFS protocol (Car + MFS; *n* = 12), saline without treatment (saline, *n* = 10), and saline treated using MFS protocol (saline + MFS, *n* = 11). Animals from the Car and Car + MFS groups were injected with 50 μL of carrageenan (Sigma, St. Louis, MO, United States) at 3% (diluted in saline 0.9%). Saline and saline + MFS groups were injected only with 50 μL of saline 0.9%.

### Procedures

#### Anesthesia and Inflammation Protocols

All animals were sedated with an intraperitoneal injection of ketamine (90 mg/kg of animal weight) and xylazine (10 mg/kg of animal weight). The animals were positioned in prone position and had their back shaved with an electric shaver and Veet hair removal cream. The local needle insertion was defined as follows: two diagonal lines were drawn starting at each coxofemoral joint and ending at the lower back, at the point where the two lines intersected; a perpendicular line starting at the spine was traced; and then 1 cm was measured to the right side of the back. Thereby, the needle was inserted at an angle of 45° on the skin and projected to reach a deep layer of the connective tissue of the thoracolumbar region. The inflammation model was based on previous literature (Corey et al., 2012). The animals from the Car and Car + MFS groups were injected with 50 μL of Carrageenan (Sigma, St. Louis, MO, United States) diluted to 3% in saline 0.9%. The animals from saline and saline + MFS groups were only injected with 50 μL of saline 0.9%. The inflammation protocol has been previously trained in animal cadavers to make sure that carrageenan/saline would reach the inner layer of the connective tissue of thoracolumbar region.

The connective tissue of the thoracolumbar region was identified using the Evans blue method. A local injection of Evans blue associated with dissection was used. Careful examination revealed that three different layers could be differentiated in the lumbar region of the animals: (i) an outer layer, immediately under the skin, consisting of loose connective tissue; (ii) a middle layer, containing thick bundles of collagen fibers that run obliquely to the spine; and (iii) an inner layer, consisting of a thin sheet of parallel collagen fibers oriented perpendicularly to the spine. This revealed that traces of Evans blue were visible in the fascial layers but not in the adjacent muscles [the paravertebral (PV) muscles and gluteus muscles]. It was concluded that the injection protocol of this study reached the middle layer as well as inner layer, in contrast to the outer layer or the underlying musculature.

#### MFS Protocol

The Car + MFS and saline + MFS mice were treated with the MFS by a researcher blinded to the experimental protocol, a physiotherapist with 15 years of practice in manual therapy and 6 months of previous training in animal experimentation. The MFS protocol was applied at 6, 24, and 48 h after the inflammation procedure, which summed three interventions during the acute phase of inflammation, with each intervention lasting 20 min. During each MFS session, the animals in the treated groups were sedated with isoflurane at 2% and oxygen at 100% and the animals in the non-treated groups were sedated as described for 20 min but did not receive treatment to act as control groups.

**FIGURE 1:**
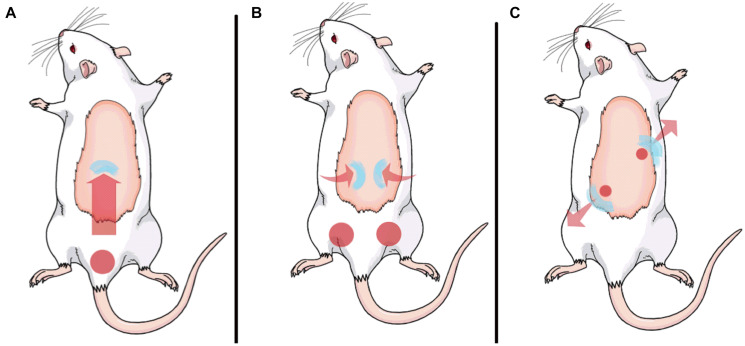
MFS protocol was subdivided into three parts. Part 1 is represented in panel **(A)** (duration of 3 min); part 2 is represented in panel **(B)** (duration of 10 min); and part 3 is represented in panel **(C)** (total duration of 7 min, with each diagonal lasting 3.5 min).

The MFS protocol is illustrated in Figure 1. The manipulation protocol used in this study, which we had described as “myofascial reorganization” in a previous investigation (Sinhorim et al., 2019), targets the fascial system. Video recordings of the specific treatment practiced in this study were evaluated by seven international experts in manual myofascial therapy, different teaching experts in manual therapy. Based on their evaluation, the specific manual treatment used in this study can be best described as a manual connective tissue manipulation expressing a strong similarity with the Rolfing method of manual therapy (Jêdrzejewski et al., 2020). Nevertheless, it included significant modifications from the original method (which was developed for humans) and does not reflect the full depth and complexity of that method. This protocol was based on previous studies about mice anatomy and dissection of the fascial complex of mice lumbar region, with the purpose of preserving the technique objectives applied to humans, but attempting to find the most appropriate correspondence between the characteristics of human treatment when applied to mice. It was applied as follows:

Moment 1: preparatory moment, duration of 3 min. With the animal in prone position, the physiotherapist anchored the inferior insertion of the fascial complex of the lumbar region (close to the tail) with one finger, and with other finger, the therapist performed continuous pressure for 45°, a shear load, in the superior insertion in the cranial direction (Figure 1A) to promote a soft stretch of connective tissue, but not a muscular one.
Moment 2: straight technique, duration of 10 min. With the animal still in the prone position, the physiotherapist anchored the inferior insertion area of the connective tissue of the thoracolumbar with two fingers at the level of the animal’s iliac, right and left side, and with two other fingers at the superior insertion, the therapist applied a tensile loading charge toward the back and the animal’s skull (Figure 1B).
Moment 3: diagonal technique, duration of 7 min. The animal was carefully positioned in supine position on the hands of the physiotherapist. Then, the therapist’s index fingers were at one side and the ring fingers at the other side of the animal’s lumbar spine, in order to contemplate all the fascial complex of the lumbar region. The therapist performed a diagonal point of pressure, with a compressive load, at first with one index finger and one ring finger, and later exchanging the pressure to the other index and ring fingers, thereby forming an X on the animal’s fascial complex of the lumbar region (Figure 1C), by that promoting a stretch of the tissue following a diagonal direction on the animal back. Each diagonal was sustained in separated moments.

The animals were euthanized at 72 h post-carrageenan/saline injection on connective tissue of the thoracolumbar region, and the tissue samples were collected.

### Procedure for Collecting Tissue Samples

The animals were euthanized with an overdose of intraperitoneal anesthesia (ketamine 180 mg/kg and xylazine 20 mg/kg). The tissue samples from the connective tissue of the thoracolumbar region and underlying PV muscle and gluteus muscle of the right side of each animal were collected by dissection and stored at −80°C on a freezer for posterior analysis. The dissection was made as follows: samples were carefully detached from the underlying muscle close to the spinous processes L4–L5 using surgical scissors. The subcutaneous tissue and its three layers were included in the sample without distinction. And then, PV muscle and gluteus samples were separated from other muscles and connective tissues. Samples were stored inside 2-mL Eppendorf microtubes.

### Western Blot Analysis

The samples were sprayed with a lysis buffer [100 mM Na_2_VO_4_, 100 mM phenylmethylsulfonyl fluoride, and a 1% protease inhibitor cocktail (P8340) (Sigma-Aldrich Co., LLC, St. Louis, MO, United States)] in T-Per (Tissue Protein Extraction Reagent; Thermo Scientific, Rockford, IL, United States). The homogenates were centrifuged at 6,000 rpm for 20 min at 4°C. The supernatant was obtained, and a separate aliquot of the protein in each sample was determined using the Bradford method (Bradford, 1976). The sample buffer [20% glycerol, 14.4 mM mercaptoethanol, 0.1% of bromophenol blue, Tris 0.2 M HCl, and 10% of sodium dodecyl sulfate (SDS)] was added at a ratio of 1:6 to the remainder of the supernatant. All the samples were boiled (95°C for 5 min) and stored at −80°C until electrophoresis analysis.

The proteins were separated using electrophoresis in a separation gel [8% acrylamide, 0.2% *bis*-acrylamide, 375 mM Tris, 0.1% SDS, 0.06% tetramethylethylenediamine (TEMED), and 0.04% ammonium persulfate] and an entry gel (4% acrylamide, 0.09% *bis*-acrylamide, 125 mM Tris, 0.1% SDS, 0.08% TEMED, and 0.03% ammonium persulfate). The samples (50 μg/well) and the molecular weight standard (Precision Plus Protein Standards, Kaleidoscope; Bio-Rad, Hercules, CA, United States) were applied to the gels, and the electrophoresis was performed at 90 V for approximately 3 h using a running buffer. After the electrophoresis, proteins were electrotransferred into polyvinylidene difluoride membranes (90 V, 90 min, and 4°C), which were blocked for 1 h at room temperature (25°C) with a 5% non-fat dry milk prepared in TBS-T (Tris-buffered saline, pH 7.4; concentration in mmol/L: 20 Tris–HCl, 137 NaCl, and 0.1% Tween 20), and incubated overnight at 4°C with primary antibodies to mouse Ly-6G, mouse F4/80, and mouse nitric oxide synthase 2 (NOS-2) at a ratio of 1:1,000 (R&D Systems Inc., Minneapolis, MN, United States), or actin–horseradish peroxidase (HRP) at a ratio of 1:50,000 (Sigma-Aldrich Co., St. Louis, MO, United States). After washing, membranes were incubated with peroxidase-conjugated secondary antibodies anti–immunoglobulin G at a ratio of 1:5,000 (Cell Signaling Technology, Danvers, MA, United States) (except for anti–actin-HRP) for 1 h at room temperature. Then, the membranes were exposed to HRP substrate (Thermo Fisher Scientific Inc., Rockford, IL, United States), and immune complexes were visualized using chemiluminescence and the iBright CL1000 System (Thermo Fischer Scientific Inc., Rockford, IL, United States). The bands were quantified using the densitometry software from the manufacturer.

### Enzyme-Linked Immunosorbent Assay

For determination of TGF-β1, TNF, IL-4, IL-6, and monocyte chemoattractant protein 1 (MCP-1) levels, a 100 μL of protein in each sample were used. These cytokine concentrations analyses were performed using the Duo Set enzyme-linked immunosorbent assay Kits (R&D Systems, Minneapolis, MN, United States) according to the manufacturer’s instructions. The values obtained were estimated by interpolating data using a standard curve for each cytokine using a colorimetric assay, which was measured at 450 nm (correction at 540 nm) in a spectrophotometer (Perlong DNM-9602, Nanjing Perlove Medical Equipment Co., Nanjing, China). The values obtained were expressed in picograms per milliliter.

### Statistical Analysis

The data were processed using GraphPad Prism 6 software. The Shapiro-Wilk test was applied to verify the data distribution. The data were analyzed using two-way analysis of variance (ANOVA) with Tukey *post hoc* test or using non-parametric Kruskal–Wallis with Dunn *post hoc* test, when adequate. The significance level was adjusted at 5% (*p* < 0.05), and the values were expressed as mean ±standard deviation (SD) for normal data, or median and interquartile interval for non-normal data.

## Results

### Tissue Cytokines and Proteins in the Connective Tissue of the Thoracolumbar

**FIGURE 2:**
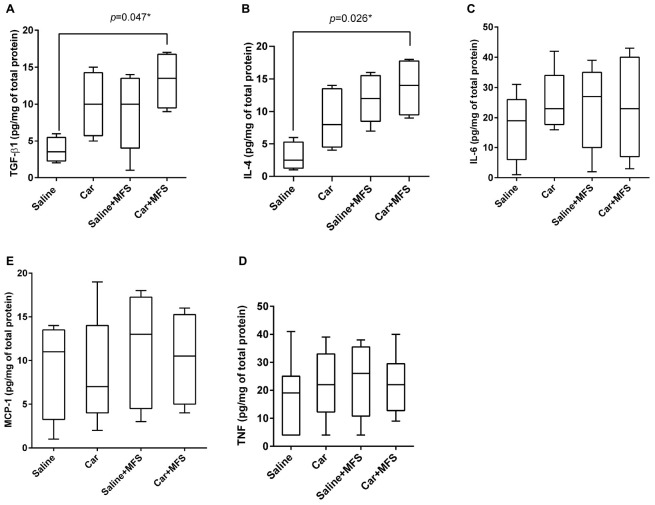
Effect of MFS on cytokine and protein levels of mice right side TA: TGF-β1 **(A)**, IL-4 **(B)**, IL-6 **(C)**, TNF **(D)**, and MCP-1 **(E)**. Levels are represented as median (interquartile interval) by group (saline, *n* = 10; Car, *n* = 11, saline + MFS, *n* = 11, and Car + MFS, *n* = 12). Data were analyzed using the Kruskal–Wallis test, with Dunn *post hoc* test and a significance level of 5% (*p* < 0.05). *There was a significant difference between saline and Car + MFS for TGF-β1 (*p* = 0.047*) **(A)** and IL-4 (*p* = 0.0265) **(B)**.

In the connective tissue of the thoracolumbar region, significantly higher levels of TGF-β1 (*p* = 0.047) and IL-4 (*p* = 0.026) were found in the Car + MFS group when compared with the saline group (Figures 2A,B, respectively). No difference was found between groups for IL-6 (*p* = 0.389), TNF (*p* = 0.555), and MCP-1 (*p* = 0.845) (Figures 2C–E, respectively) (Table 1).

**TABLE 1:** Inflammatory mediator levels in the connective tissue of the thoracolumbar region per group.

	**Saline**		**Car**		**Saline + MFS**		**Car + MFS**		***p* values**
	**Median**	**Q3–Q1**	**Median**	**Q3–Q1**	**Median**	**Q3–Q1**	**Median**	**Q3–Q1**	
TGF-β1	0.77	0.54	1.85	2.52	1.97	2.95	3.18	3.68	0.048*
TNF	0.63	4.36	0.86	2.44	1.12	2.41	0.92	2.72	0.555
IL-4	0.49	2.07	3.37	6.86	4.8	7.04	7.35	11.38	0.009**
IL-6	0.52	1.84	0.76	11.17	1.16	4.16	0.82	18.24	0.389
MCP-1	51.2	132.6	34.5	234.8	110	208.9	46.6	155.5	0.845
Ly-6G	4.42	7.12	1.23	3.40	3.89	13.61	1.62	3.62	0.437
F4/80	0.88	2.44	0.523	1.63	1.5	4.103	0.343	3.00	0.001**
NOS-2	0.95	8.02	0.3	1.65	1.18	3.612	0.293	0.65	0.002**

Mediator levels on the connective tissues of the thoracolumbar region per group are represented by median and interquartile interval (Q3–Q1). Kruskal–Wallis with Dunn *post hoc* was used for statistical analysis for all variables. *There was significant (*p* < 0.05) difference for TGF-β1, IL-4, Ly-6G, and NOS-2. Differences were found between saline vs. Car + MFS (*p = 0.047) for TGF-β1; saline vs. Car + MFS (*p = 0.026) for IL-4; Car vs. saline + MFS (**p = 0.003) and saline + MFS vs. Car + MFS (*p = 0.0337) for Ly-6G; saline vs. Car + MFS (*p = 0.0337) and saline + MFS vs. Car + MFS (*p = 0.0195) for NOS-2.

### Neutrophil (Ly-6G) and Macrophage (F4/80) Membrane Proteins the Connective Tissue of the Thoracolumbar Region

**FIGURE 3:**
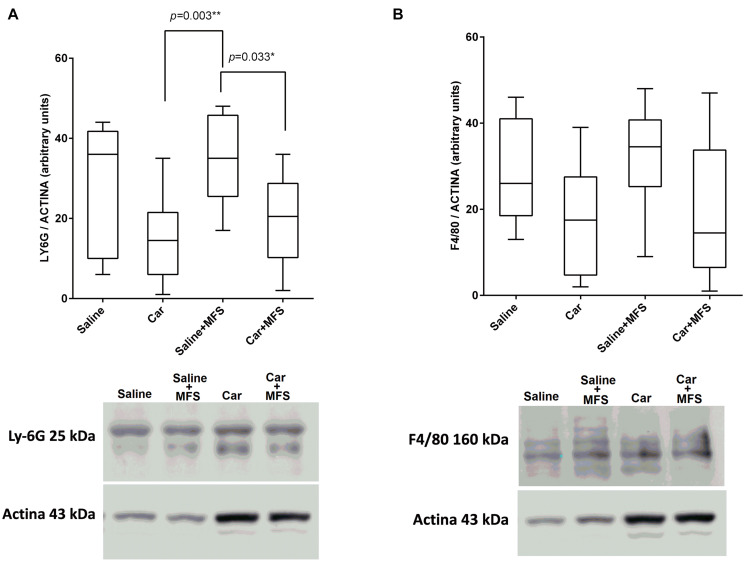
Effect of MFS on cytokine and protein levels of the right side of TA on Ly6G **(A)** and F4/80 **(B)**. Levels are represented as median (interquartile interval) by group (saline, *n* = 10; Car, *n* = 11, saline + MFS, *n* = 11, and Car + MFS, *n* = 12). Data were analyzed using the Kruskal–Wallis test, with Dunn *post hoc* test and a significance level of 5% (*p* < 0.05). *There was a significant difference between the Car and saline + MFS groups (*p* = 0.0033**) and between the saline + MFS and Car + MFS groups (*p* = 0.0337*) for Ly-6G protein **(A)**. No difference was found between treated and non-treated animals on terms of F4/80.

There was no significant difference for the Ly-6G protein (Figure 3A) present on the neutrophil membrane when comparing the saline with the Car group (*p* = 0.086), the saline with saline + MFS group (*p* > 0.999), the saline with the Car + MFS group (*p* = 466), and/or the Car with the Car + MFS group (*p* > 0.999). Interestingly, Ly-6G protein levels were significantly lower in the Car + MFS when compared to saline (*p* = 0.003) or saline + MFS (*p* = 0.033) groups. No difference was found for the F4/80 protein presented on the macrophage membrane when comparing the saline and Car groups (*p* = 0.437), the saline and saline + MFS groups (*p* > 0.999), the saline and Car + MFS groups (*p* = 0.652), the Car and Car + MFS groups (*p* > 0.999), or the saline + MFS and Car + MFS groups (*p* = 0.105) (Figure 3B).

### NOS-2 on the Connective Tissue of the Thoracolumbar Region

**FIGURE 4:**
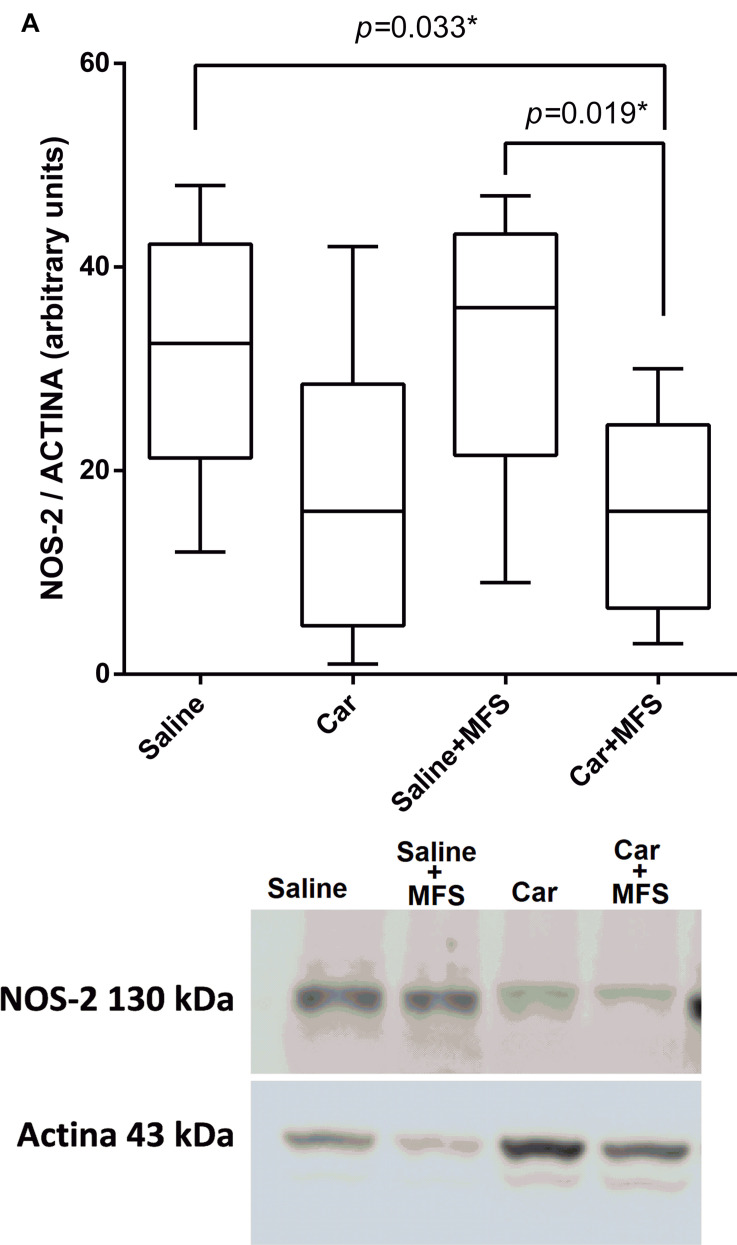
Effect of MFS on NOS-2 levels in the right side of the TA. Levels are represented as median (interquartile interval) by group (saline, *n* = 10; Car, *n* = 11; saline + MFS, *n* = 11; and Car + MFS, *n* = 12). Data were analyzed using the Kruskal–Wallis test, with Dunn *post hoc* test and a significance level of 5% (*p* < 0.05). ^∗^There was a significant difference between the saline and Car + MFS (*p* = 0.0337^∗^) and Car groups (*p* < 0.0033) and between the saline + MFS and Car + MFS groups (0.0195^∗^).

As shown by Western blot analysis, NOS-2 levels (Figure 4), the enzyme responsible for the synthesis of nitric oxide (NO), were not different between the saline and Car groups (*p* = 0.093), the saline and saline + MFS groups (*p* > 0.999), the Car and Car + MFS groups (*p* > 0.999), or the Car and saline + MFS groups (*p* > 0.056). The levels of NOS-2 were significantly lower in the Car + MFS group when compared with the saline (*p* = 0.033) and the saline + MFS (*p* = 0.019) groups.

### Tissue Cytokines in PV and Gluteus Muscles

**FIGURE 5:**
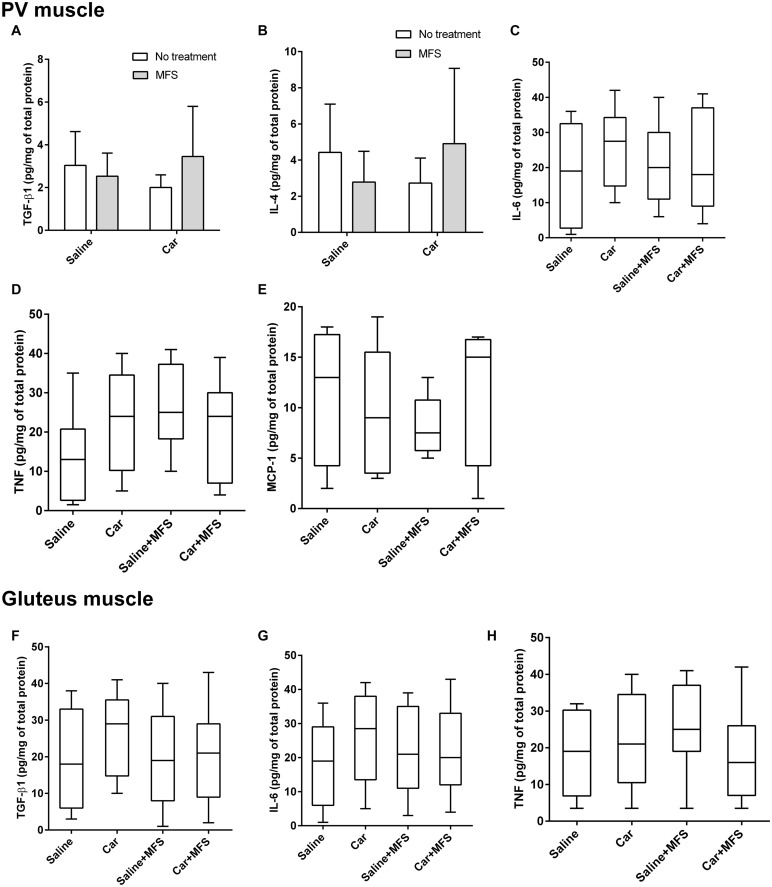
No significant difference was found in the protein and cytokine levels of PV and gluteus muscles. TGF-β1 **(A)**, IL-4 **(B)**, IL-6 **(C)** TNF **(D)**, and MCP-1 **(E)** are representative of the PV of saline (*n* = 10), saline + MFS (*n* = 11), Car (*n* = 11), and Car + MFS (*n* = 12). TGF-β1 **(A)** and IL-4 **(B)** are represented as mean ± SD and were analyzed using ANOVA with Tukey *post hoc* test. IL-6 **(C)**, TNF **(D)**, and MCP-1 **(E)** are presented as median (interquartile interval) and were analyzed using Kruskal–Wallis with Dunn *post hoc* test. Gluteus TGF-β1 **(F)**, IL-6 **(G)**, and TNF **(H)** of saline (*n* = 10), saline + MFS (*n* = 11), Car (*n* = 11), and Car + MFS (*n* = 12) are shown as medians (interquartile intervals) and were analyzed using Kruskal–Wallis with Dunn *post hoc* test. On the PV muscle, there was no difference between groups for TGF-β1 (*p* > 0.1673) **(A)**, IL-4 (*p* > 0.1241) **(B)**, IL-6 (*p* > 0.7023) **(C)**, TNF (*p* > 0.1437) **(D)**, and MCP-1 (*p* = 0.68) **(E)** levels. In the right gluteus muscle, no difference was found between groups for the TGF-β1 (*p* = 0.6329) **(F)**, IL-6 (*p* = 0.48) **(G)**, or TNF (*p* = 0.3750) **(H)**.

In the PV muscle, there was no difference in the levels of TGF-β1 (*p* = 0.167) (Figure 5A), IL-4 (*p* = 0.124) (Figure 5B), IL-6 (Figure 5C) (*p* = 0.480), TNF (*p* = 0.144) (Figure 5D), or MCP-1 (*p* = 0.686) (Figure 5E) between groups (*p* > 0.05) (Table 2). In the right gluteus muscle, no difference was found between groups for TGF-β1 (*p* = 0.632) (Figure 5F), IL-6 (*p* = 0.485) (Figure 5G), or TNF (*p* = 0.375) (Figure 5H) (Table 3).

**TABLE 2:** Inflammatory mediator levels in PV muscle per group.

	**Saline**		**Car**		**Saline + MFS**		**Car + MFS**		***p* values**
	**Mean**	**SD**	**Mean**	**SD**	**Mean**	**SD**	**Mean**	**SD**	
TGF-β1	3.03	±1.59	2.00	±0.59	2.53	±1.08	3.45	±2.35	0.167
IL-4	4.43	±1.39	2.72	±1.39	2.78	±1.70	4.91	±4.17	0.124
	Median	Q3–Q1	Median	Q3–Q1	Median	Q3–Q1		Q3–Q1	*p* values
TNF	0.99	5.74	2.41	7.45	2.47	8.579	2.44	7.43	0.144
IL-6	1.84	3.82	3.03	9.21	2	5.755	1.77	8.86	0.480
MCP-1	33.4	111.1	23.1	216	20.5	18.1	46	50	0.686

*Mediator levels on PV muscle per group are represented. TNF, IL-6, and MCP-1 levels were analyzed using Kruskal–Wallis with Dunn *post hoc* test and are represented by median and interquartile interval (Q3–Q1). TGF-β1 and IL-4 were analyzed using ANOVA with Tukey *post hoc* test and are represented by mean and SD. No significant differences were found between groups for all variables.*

**TABLE 3:** Inflammatory mediator levels in gluteus muscle per group.

	**Saline**		**Car**		**Saline + MFS**		**Car + MFS**		***p* values**
	**Median**	**Q3–Q1**	**Median**	**Q3–Q1**	**Median**	**Q3–Q1**	**Median**	**Q3–Q1**	
TGF	1.45	8.65	3.51	12.09	1.51	11.58	1.82	18.89	0.632
TNF	1.09	3.49	1.47	15.8	1.89	37.3	0.794	47.6	0.375
IL-6	0.88	3.57	2.06	6.99	1.26	4.55	0.997	7.99	0.485

*Mediator levels on gluteus muscle per group are represented by median and interquartile interval (Q3–Q1). Kruskal–Wallis with Dunn *post hoc* was used for statistical analysis for all variables. No differences were found between groups.*

## Discussion

Significantly higher levels of TGF-β1 and IL-4 were found in the connective tissue of the thoracolumbar region in the animals with inflamed tissue and subsequently treated with MFS when compared with the group of animals administered with saline. The levels of Ly-6G protein and NOS-2 were significantly lower in the inflamed and treated with MFS group rather than in the saline group.

The excess of TGF-β1 has been associated with fibrosis formation in several models of treatment of chronic inflammation (Bouffard et al., 2008; Bove et al., 2016; Gyorfi et al., 2018), as it has been for IL-4 (Trautmann et al., 1998, 2000). In models of chronic inflammation, different manual therapies, such as joint mobilization and upper extremity skin traction (Bove et al., 2016, 2019), tissue stretching *ex vivo* (Bouffard et al., 2008) and mobilization of the fibrotic scar (Altomare and Monte-Alto-Costa, 2018) are related to a positive improvement of this fibrotic change in the connective tissue. Among the positive changes, lower deposition of type I collagen and TGF-β1 in connective tissue (Bouffard et al., 2008; Bove et al., 2016), lower deposition of collagen and fewer presence of neutrophils and macrophage around median nerve (Bove et al., 2019), improvement of skin mobility, and reduction of ECM stiffness (Altomare and Monte-Alto-Costa, 2018) have been observed.

However, during acute inflammation, the finding of an elevation in the levels of TGF-β1 together with IL-4 was not expected. Yet we observed the enhancement of these two mediators without elevating the levels of proinflammatory cytokines, such as TNF and IL-6 in the connective tissue of the thoracolumbar region. In this way, the TGF-β1 together with IL-4 may be acting as proresolving cytokines. This enhancement could be related to an effect of MFS on the connective tissue, and it could be associated with proresolutive environment, which was promoted by the mechanical stimulation.

Berrueta et al. (2016) suggested that mechanical stimulation has an effect on inflammation resolution in connective tissue. In their study, a mechanical stimulus induced by a stretching had promoted a proresolutive environment in a rat model (Berrueta et al., 2016). The *in vivo* results showed fewer neutrophil cells and a widespread increase in proresolving mediators such as RvD1 and RvD2 in the stretched rats compared with the non-stretched (Berrueta et al., 2016).

By using a similar approach (stretching a connective tissue) in a breast cancer mice model (Berrueta et al., 2018), a daily gentle stretching for 10 min for 4 weeks led to significantly slow tumor growth and a 52% smaller tumor size in the stretched mice compared with the non-stretched mice; that said, the reduction in the tumor could be associated with the cytokine levels, according to the authors. They did not observe individual group differences but reported that IL-10, IL-2, IL-6, TNF-α, and interferon γ were up-regulated in the stretch group. The tumor levels of lipid-derived specialized proresolution mediators, RvD1 and RvD2, which promote the natural resolution of inflammation, were significantly greater in the stretched compared to the non-stretched mice (Berrueta et al., 2018). In contrast to their results, we did not observe up-regulation of TNF-α or IL-6, although in both studies (Berrueta et al., 2016, 2018) manual therapy was not applied as treatment as well done in our study. Our findings may be also related to a proresolutive state facilitated by mechanical stimulation of the connective tissue during acute inflammation.

The enhancement of IL-4 and TGF-β may favor the recruitment of macrophage type 2 (M2), an anti-inflammatory macrophage phenotype. In skeletal muscle, during the first postinjury hours, there is a recruitment of circulating monocytes, which can enter the tissue and differentiate into inflammatory macrophages type 1 (M1). At the time the M1 macrophages are exposed to an inflammatory environment and perform the muscle fiber debris phagocytosis, they are switching into the M2 profile, and when they are stimulated by IL-4, these M2 (anti-inflammatory) macrophages start to produce TGF-β1 (Arnold et al., 2007). These anti-inflammatory macrophages (M2) are stimulated in the presence of mediators such as IL-4 and TGF-β (Fraternale et al., 2015; Sang et al., 2015), whereas M1 macrophages are polarized by TNF and IL-6. Additionally, IL-4 is associated with the transformation of M1 into M2 macrophages (Mantovani et al., 2004). Moreover, it may be possible that TGF-β1 and IL-4 interact with one another to provide a proresolutive environment, once the increasing concentration of the combination of TGF-β1 and IL-4 diminishes the production of other ILs, including IL-4 as demonstrated by Dardalhon et al. (2008).

Although we observed an increase in TGF-β1 and IL-4 at 72 h after inflammation, there was no significant increase in MCP-1 or F4/80 protein in the connective tissue of the thoracolumbar region. It is presumed that the massive presence of F4/80^+^ cells occurs from the fourth day onward, as observed by Brigitte et al. (2010). In this way, it is possible that F4/80^+^ cells would be detectable only after that period. It has been shown that in the carrageenan inflammation model, the presence of mononucleated cells, such as macrophages, starts after 48 h (Martin et al., 1994). Brigitte et al. (2010) did not use carrageenan, but notexin, and they observed a peak of monocyte/macrophage cells (F4/80^+^ cells) on the epimysium on the fourth day after inflammation. These cells remained elevated in number until the 12th day on the epimysium/perimysium of the tibial and PV muscles of rats (Brigitte et al., 2010).

There was significant decrease in Ly-6G expression in the Car + MFS group when compared to the saline + MFS, as there was significant decrease in its expression in Car + MFS group when compared with saline group. There was no difference between the Car and the Car + MFS group. The animals that received carrageenan injection treated with MFS showed higher levels of IL-4, which inhibits chemotaxis and migration of neutrophils (Saleem et al., 1998; Impellizzieri et al., 2019) to the point that systemic inhibition of IL-4 increases the influx of CD11b^+^Ly6G^+^ cells (neutrophils) during infection (Woytschak et al., 2016). We observed lower levels of Ly6G in the Car and the Car + MFS groups, but this finding could be related to the enhancement of IL-4. There was no significant difference between the saline + MFS and the Car groups for IL-4, whereas there was difference between the saline + MFS and the Car groups for Ly6G.

Lower levels of Ly6G^+^ cells could be explained by NOS-2 [induced NOS (iNOS)] reduction, as Salvemini et al. (1996) demonstrated that iNOS inhibition also attenuates neutrophil infiltration during carrageenan inflammation of a paw in a mouse model. Our findings differ from those of Salvemini et al., as there was significant difference between the saline and the Car + MFS groups for Ly6G, but not for NOS-2, thereby the presence of a few neutrophils could be linked to the lack of macrophages, according to Rigamonti et al. (2013), in acute muscle injury. Although, iNOS is produced early by neutrophils, by day 3 after induction of inflammation, iNOS is produced by macrophages. We evaluated those NOS-2 levels at the third day.

Salvemini et al. (1996) identified that neutrophils were not the only source of iNOS, as macrophage-like cells also expressed the enzyme at their membranes. The expression of iNOS was detected at 6 h after paw inflammation in the carrageenan mice model, and the maximal expression of iNOS occurred at 10 h after inflammation. Immunoreactivity for iNOS was detected in macrophage-like cells, but not in neutrophils, which suggests that resident macrophages or infiltrated monocytes can be a source of NO as produced by iNOS (Salvemini et al., 1996); however, our data did not show an increase in F4/80 protein (macrophages). In addition, the monocytes derived from the blood have shown their peak on connective tissue around the muscle connective tissue (epimysium/perimysium) by the fourth day after inflammation (Brigitte et al., 2010); this period did not coincide with the period evaluated in our study. Under these circumstances, it is not likely that MFS has a direct or little effect on iNOS levels.

It is to be noted that iNOS inhibition, as shown by Liu et al. (2015), on the fourth day of rats inflamed with notexin and treated with an inhibitor of iNOS, provoked a major infiltration of F4/80^+^ cells when compared to the group inflamed/without inhibitor agent. Liu et al. (2015) concluded that NO augments mRNA expression of TGF-β1 and IL-10 and decreases MCP-1, TNF, IL-6, and proinflammatory cytokines. In our study, there was an increase in TGF-β1 without significant changes in the F4/80^+^ levels on the third day after inflammation. In addition, there was no significant change in MCP-1 between groups. The TGF-β1 levels may have suffered some effect from MFS while inflammatory cells may have not.

Another important fact to be noted is that MFS did not cause an increase or spread of inflammation provoked by carrageenan, as there was no significant increase of proinflammatory mediators (TNF and IL-6) at 72 h after carrageenan injection. This agent alone increased TNF and IL-6 levels in the first hour after inflammation when compared with the saline group (Loram et al., 2007b; Annamalai and Thangam, 2017). However, in this model, TNF and IL-6 levels were not different at 24 h between rodents injected with saline or carrageenan (Loram et al., 2007a,b). In addition, a lack of TGF-β1, IL-4, MCP-1, TNF, or IL-6 in the underlying paraspinal muscles or gluteus in either treated or untreated animals suggests that the MFS protocol does not cause spread of proinflammatory factors to the surrounding tissue.

It is to be noted that no differences were found between groups for ILs and inflammatory mediators, including IL-4 and TGF-β1, on underlying muscles, as MFS is specifically applied on the connective tissue of the thoracolumbar. Therefore, MFS may be safe to be applied in the management of early stages of acute inflammation in orthopedic conditions, as it may have a proresolutive effect on inflamed tissue without significant recruitment of inflammatory cells or enhancement of proinflammatory mediators.

In conclusion, there may be a potentiating effect on the production of TGF-β1 and IL-4, anti-inflammatory factors, without increasing MCP-1, TNF, and IL-6 proinflammatory factors in carrageenan model. MFS had no direct effect on Ly6G^+^ or F4/80^+^ cells, whereas Ly6G^+^ may have little or no association with IL-4 raising. NOS-2 reduction may be linked to the lack of increase in F4/80^+^ cells, which can be major producers of NOS-2 and are not yet present in the tissue at the third day in a significant amount. Also, there were no significant differences between treatment and control (saline) animals for F4/80. Therefore, NOS-2 reduction is not likely to be related to the MFS protocol. In addition, there were no significant changes in TGF-β1, IL-4, MCP-1, TNF, and IL-6 in underlying muscles. The lack of TGF-β1, IL-4, MCP-1, TNF, or IL-6 into the underlying paraspinal muscles in either treated or untreated animals suggests that the MFS protocol does not cause spread of proinflammatory factors to the surrounding tissue. MFS specifically targets the connective tissue, but further investigation is needed to observe the effects of this technique in early phases and later phases of inflammation of the connective tissue. We conclude that MFS may be a safe treatment to help manage acute inflammatory processes in orthopedic pain conditions, as it does not provoke enhancement of inflammation, but it promotes a proresolutive environment on treated connective tissue.

The differences in the function and local topographical anatomy of the thoracolumbar fascia of rats and mice to that of humans should be taken into account, because of their differences in locomotion, basic anatomy, and posture (Loram et al., 2007a). However, previous examinations by other investigations with fascial tissues from different types of mammals have revealed common adaptive physiological responses of such connective tissues in response to mechanical stimulation (Cao et al., 2015; Langevin et al., 2018; Paxton et al., 2020). It is therefore suggested that the basic findings about these physiological responses from the present study will most likely also apply to some degree to the mechanoadaptive response of the connective tissue of the thoracolumbar region of human.

## Limitations

As it is an unprecedented study, some aspects may have influenced its results: sample collection was performed at 72 h (3 days) after inflammation; during this period, a transition phase may have occurred, and an increase in inflammation preceding a chronic stage may have occurred in this model. Further research with a carrageenan model must consider the experiments using a time-course design, where carrageenan inflammation effects alone can be evaluated at 6, 24, 48, 72 h, and after the fourth day, in a way of identifying the cell and mediator profiles of this model. The detailed profile of carrageenan inflammation following both acute and chronic stages is not well established in the literature. After that, an MFS treatment using this model is suggested to be applied in the first 4 days, to compare with no MFS treatment conditions. Anesthesia with ketamine may have influenced the results, but it was chosen to avoid pain avoidance reactions by the animals; however, the substitution of ketamine by isoflurane has been suggested. However, ketamine this influence may have been minimal, as observed by Goulart et al. (2005). The author did not report any collateral effects or influence by anesthesia using ketamine and xylazine when investigating carrageenan inflammation effects in the temporomandibular joint of rats.

## Data Availability Statement

The raw data supporting the conclusions of this article will be made available by the authors, without undue reservation.

## Ethics Statement

The animal study was reviewed and approved by Ethics Committee for the Use of Animals – University of the South of Santa Catarina.

## Author Contributions

MF and LS: literature review, data collection, data analysis, and final edition. DM and RS: final edition. NM-P and GS: data collection. VH: data collection, data analysis, and final edition. GS: data analysis and final edition. All authors contributed to the article and approved the submitted version.

## Conflict of Interest

The authors declare that the research was conducted in the absence of any commercial or financial relationships that could be construed as a potential conflict of interest. The reviewer JD declared a past co-authorship with one of the authors RS to the handling editor.
